# Medial parapatellar surgical approach leads to greater loss of postural sway complexity compared to mid‐vastus approach in women undergoing total knee arthroplasty

**DOI:** 10.1002/ksa.70057

**Published:** 2025-09-09

**Authors:** Vasileios Mylonas, Stylianos Grigoriadis, Dimitris Metaxiotis, Eleftherios Kellis, Nick Stergiou, Thomas Nikodelis

**Affiliations:** ^1^ Division of Biomechanics and Research Development, Department of Biomechanics and Center for Research in Human Movement Variability University of Nebraska at Omaha Omaha Nebraska USA; ^2^ Biomechanics Laboratory, School of Physical Education & Sport Science at Thessaloniki Aristotle University of Thessaloniki Thessaloniki Greece; ^3^ 2nd Orthopaedic Department General Hospital of Thessaloniki “Papageorgiou” Thessaloniki Greece; ^4^ Laboratory of Neuromechanics, School of Physical Education & Sport Science at Serres Aristotle University of Thessaloniki Serres Greece

**Keywords:** balance, complexity, osteoarthritis, posture, variability

## Abstract

**Purpose:**

Total knee arthroplasty (TKA) is associated with acute postoperative effects that increase the risk of falls. These effects differ between the medial parapatellar (PP) and mid‐vastus (MV) surgical techniques but have not been evaluated in terms of postural sway complexity. Loss of this complexity leads to increased randomness in the center of pressure and higher fall risk. This exploratory comparative analysis addresses this knowledge gap by examining how PP and MV techniques affect postural sway complexity in women.

**Methods:**

Twenty women with osteoarthritis (OA) were randomly assigned to TKA via the PP or MV approach. Postural sway data were collected at: 1 day before the surgery (Pre), 5 days (Post), 2 weeks, and 1 month post‐surgery. We also included an age‐ and sex‐matched healthy control group (*n* = 11) with a single assessment of their postural sway. The temporal structure of variability was evaluated in terms of its complexity. We quantified the complexity of postural sway using detrended fluctuation analysis (DFA) and multi‐scale entropy (MSE).

**Results:**

The α exponent (DFA) and MSE values showed significant effects of time in both axes, with both surgical groups exhibiting a shift towards more random patterns following TKA. When compared to controls, the PP group exhibited a significantly lower α exponent, indicative of more random patterns, at 2 weeks and 1 month but not at Pre and Post, while no such differences were observed for the MV group. Lower α exponent and higher MSE values reflect reduced sway complexity.

**Conclusions:**

These findings suggest that TKA leads to loss of complexity in women during the postoperative stage, with the PP approach resulting in more pronounced reductions relative to healthy controls than the MV approach. Future studies should explore the long‐term effects of TKA on postural sway complexity and the impact of rehabilitation protocols.

**Level of Evidence:**

N/A.

**Abbreviations**
AP: anterior‐posterior
CoP: center of pressure
DFA: detrended fluctuation analysis
ML: mediolateral
MSE: multi‐scale entropy
MV: mid‐vastus
OA: osteoarthritis
PP: parapatellar
TKA: total knee arthroplasty

## INTRODUCTION

Knee osteoarthritis (OA) is a disease that causes changes in the joint articular cartilage, affecting well over 650 million individuals worldwide [16], predominantly older individuals (>60 years old) and women [72]. Common symptoms of OA include knee joint pain, limited range of motion, and reduced balance control during postural and gait tasks [15, 36, 42, 84, 94]. The overall deterioration in physical abilities is leading to an increased number of falls in knee OA patients [20]. Total knee arthroplasty (TKA) is the primary surgical procedure for treating end‐stage knee OA [8]. TKA effectively relieves pain and improves range of motion, weight loading, knee strength, proprioception and the overall functionality of patients [8]. However, the acute effects of TKA are significant, and patients showcase reduced knee strength, proprioception, and range of motion [1, 3, 4, 55, 60, 81]. Traditionally, the surgical method is the medial parapatellar (PP) approach, which involves a longitudinal section of the quadriceps tendon. Nevertheless, this approach damages the quadriceps tendon, thus compromising the extension mechanism of the knee [6]. Another frequently used technique is a minimal invasive, mid‐vastus (MV) approach, during which the surgeon surpasses the quadriceps tendon and carries out an incision on the vastus medialis muscle [26, 83, 85]. Thus, compared to PP, the knee's extension mechanism suffers less compromise. However, in both methods, the immediate effects of surgery can compromise postural stability, making patients more likely to fall in the first year after TKA [5, 10, 11].

Postural stability has been studied extensively by evaluating the postural sway fluctuations present in the center of pressure (CoP) [25, 51, 57, 65, 68, 96]. CoP fluctuations represent the individual's efforts to maintain the center of mass within the base of support through continuous corrective adjustments [91]. Traditionally, CoP time series were analyzed in terms of their speed, total length or other metrics of magnitude [12, 88]. However, these metrics disregard the temporal structure of variability or how sway fluctuations evolve over time [39, 48]. Therefore, new measures have been suggested to investigate the temporal structure of variability, allowing for a better understanding of the patterns present in postural sway [38, 43, 49, 52, 54, 87]. Similar analyses are also utilized in gait and heart rate to quantify stability and autonomic control [14, 31, 34, 50, 61, 67, 71, 74, 79, 92]. The temporal structure of the variability is evaluated in terms of its ‘complexity’, which is defined as the elaborate interactions of the multiple subcomponents of the human body that eventually produce effective control.

The term complexity describes the fractal‐like interactions that exhibit correlations and self‐similarity in biological signals [9, 21, 29]. This type of interaction is considered as an optimal state of movement variability and refers to temporal fluctuations that also display power law scaling (the frequency of oscillations is inversely proportional to their power) and is referred to as 1/f [21, 24]. Healthy human movement variability that displays 1/f power law scaling is characterized by adaptability to changes and stability to perturbations [30, 66, 77]. Ageing and/or pathology, however, can significantly alter these dynamics and result in loss of complexity and the breakdown of this structure [45, 76]. Fluctuations may present uncorrelated patterns without any persistent behaviour, resembling randomness [7, 30, 32, 37, 67, 82] or may exhibit extremely increased persistency that is reflected in repeatable behaviour, resembling Brownian motion [69, 86]. In both cases, the ability of human movement to drastically adapt to changes and perturbations is compromised [29, 76, 77]. Extensive research supports the predicted associations between movement complexity and adaptability in postural sway. Reduced complexity was associated with developmental delays and cerebral palsy [18, 19, 27, 38], where targeted interventions based on this insight helped accelerate the acquisition of sitting posture [28]. Furthermore, the complexity of postural sway variability while maintaining upright position has been associated with falls in older adults [86, 96]. Certain interventions that have targeted postural sway have been found to effectively reduce the risk of falls in older adults, also highlighting the importance of complexity assessment [47, 80, 89, 93].

Regarding knee OA, although falls are a major problem in patients with OA both before and after TKA [5, 10, 11], little to no research has been conducted to identify complexity in the postural sway variability of OA patients. Previous studies have reported a loss of postural sway complexity in OA patients compared to healthy controls, and a similar reduction between 4 and 12 weeks post‐TKA [13, 54]. Recent evidence suggests that the surgical approach may be associated with different levels of postural control deterioration [41, 55, 59]. Patients who undergo TKA using the traditional PP approach are less adaptable to perturbations compared to patients who undergo minimally invasive TKA [59]. In addition, parameters of stability during walking were different in PP patients as compared to controls, while differences were not that evident for the MV group [41]. Recently, it was found that the magnitude of postural sway increases after TKA only in a PP patient group [55]. Together, these findings suggest that the PP surgical approach compromises postural stability to a greater extent compared to the MV approach. However, epidemiological studies that investigated such incidents following TKA have not taken surgical approaches into account [5, 10, 11].

There has been limited effort to identify the complexity in postural sway variability after TKA. Clark et al. reported decreased complexity of postural sway variability in TKA patients at 12 weeks compared to 4 weeks post‐surgery [13], but the absence of pre‐surgery data and a healthy control group limits the interpretation of those findings. Furthermore, given the established link between complexity, adaptability, and fall risk, understanding how TKA impacts sway complexity is essential. To strengthen interpretation and determine whether any observed reductions in complexity reflect pathological deviations rather than normal postoperative adaptation, postural sway recordings from age‐matched controls should be considered. This comparison will provide a normative reference point, allowing us to better assess the clinical relevance of complexity changes following TKA and potentially guide future fall prevention strategies.

Therefore, the purpose of the study is to identify how the complexity of postural sway variability is altered in patients who undergo TKA. More specifically, we examined the acute effects of a traditional (PP) and a minimally invasive (MV) TKA approach on the complexity of postural sway variability. Based on the available literature, we hypothesized that both patient groups would demonstrate reduced complexity following TKA compared to pre‐surgery levels. We also hypothesized that patients who undergo TKA using the PP approach would demonstrate greater reduction as compared to patients who undergo TKA using the MV approach. Additionally, we sought to compare the postural sway complexity of the two patient groups with age‐ and gender‐matched healthy controls. We hypothesized that the postural sway complexity of the control group would be higher compared to the PP and MV groups prior to the surgery, and that the PP group would deviate more substantially from healthy controls than the MV group during postoperative recovery.

## METHODS

### Participants

This study employed an exploratory comparative observational design to analyze data from two patient groups of older female adults as well as from a third group of healthy controls, which was used for normative comparison. Individuals of the two patient groups suffered from severe, end‐stage OA and underwent TKA. Ten patients (age: 70.6 ± 9.2, height: 160.9 ± 7.1 cm, weight: 86.5 ± 13.0 kg) underwent surgery using the traditional PP approach (first group), while 10 patients (age: 66.46 ± 6.1, height: 160.3 ± 5.0 cm, weight: 86.9 ± 10.8 kg) underwent surgery using the MV approach (second group). Participants were randomly assigned to MV or PP groups. All patients underwent a standardized postoperative rehabilitation programme that included muscle strengthening and joint mobilization exercises. Additionally, all patients received the same standardized analgesic protocol consisting of intravenous Paracetamol (1 g, three times daily) and Tramadol (100 mg, twice daily) for the first three days. This dataset was also utilized in a previous study by our research group with a different purpose [55]. The original data collection was approved by the hospital's ethics committee (approval number: 2022‐В2015–232), and all participants provided written informed consent. For comparative purposes, we also included a group of age‐ and sex‐matched healthy controls. Posturographic data for this group were obtained from a publicly available repository [70]. The control group consisted of eleven healthy females (age: 68.5 ± 5.3 years, height: 154.4 ± 5.7 cm, weight: 61.3 ± 6.5 kg) with no history of knee pathology. Ethical approval and informed consent procedures for the healthy control data were already in place at the time of original data collection (approval number: 842529/2014).

To enhance methodological transparency, this study adheres to the STROBE (Strengthening the Reporting of Observational Studies in Epidemiology) guidelines for comparative observational studies. Although the original data were collected as part of a prospective study, this analysis represents a secondary exploratory investigation focused on postural sway complexity. Randomization was performed using a simple binary random number generator without stratification or blocking. Allocation concealment and blinding of outcome assessors or data analysts were not performed, and this limitation is acknowledged and discussed in the manuscript. All participants followed standardized surgical, rehabilitation and analgesic protocols, minimizing potential confounding from treatment heterogeneity. The ethical procedures, data flow and group comparability are fully documented in this manuscript to comply with recommended best practices in orthopaedic research reporting [62].

A summary of the shared methodological procedures is provided below. More details can be found in our previous publication [55] and the original source of the healthy control data [70].

### Surgical approaches and data collection

The same orthopaedic surgeon, experienced in both PP and MV approaches, performed all surgical operations. In both techniques, the incision in the skin and subcutaneous tissue is medium and 12–15 cm long. During the PP approach, the incision was made at the medial border of the quadriceps tendon, originating approximately 5 cm medial to the top of the patellar bone. From there, the incision was extended peripherally to the medial border of the patellar bone and the patellar tendon until the tibial tuberosity. Following a medial PP bursectomy, the patella was everted outward, and the knee was flexed as much as possible. During the MV approach, the incision was made approximately parallel with the vastus medialis oblique muscle fibres until it reached the upper medial side of the patellar bone. There, the incision was extended peripherally to the medial border of the patellar bone and tendon, similar to the PP approach [33].

Posturographic data were collected one day before the surgery (Pre), 5 days (Post), 2 weeks and 1 month after the surgery. Patients were asked to stand still with open eyes for 30 s in a dual‐plate portable force platform (K‐Plates, @Kinvent Biomecanique; 330 × 175 mm each) set to measure at 75 Hz. Data were downsampled to 25 Hz to avoid inflated persistency caused by oversampling [95]. Force plates recorded the ground reaction force, from which the CoP was then calculated [90].

Regarding the control group, individuals were asked to stand still with eyes open for 60 s in a dual‐plate force platform (OPT400600‐1000, AMTI; 400 × 600 mm each), which was set to record data at 100 Hz. We used only the first 30 s in order to match the total duration of the data collection in our TKA groups. The CoP was calculated from the ground reaction force data of the force platform for all trials, which were downsampled to 25 Hz to align with the sampling frequency used in the data collections of our patient groups.

### Data analysis

**Figure 1:**
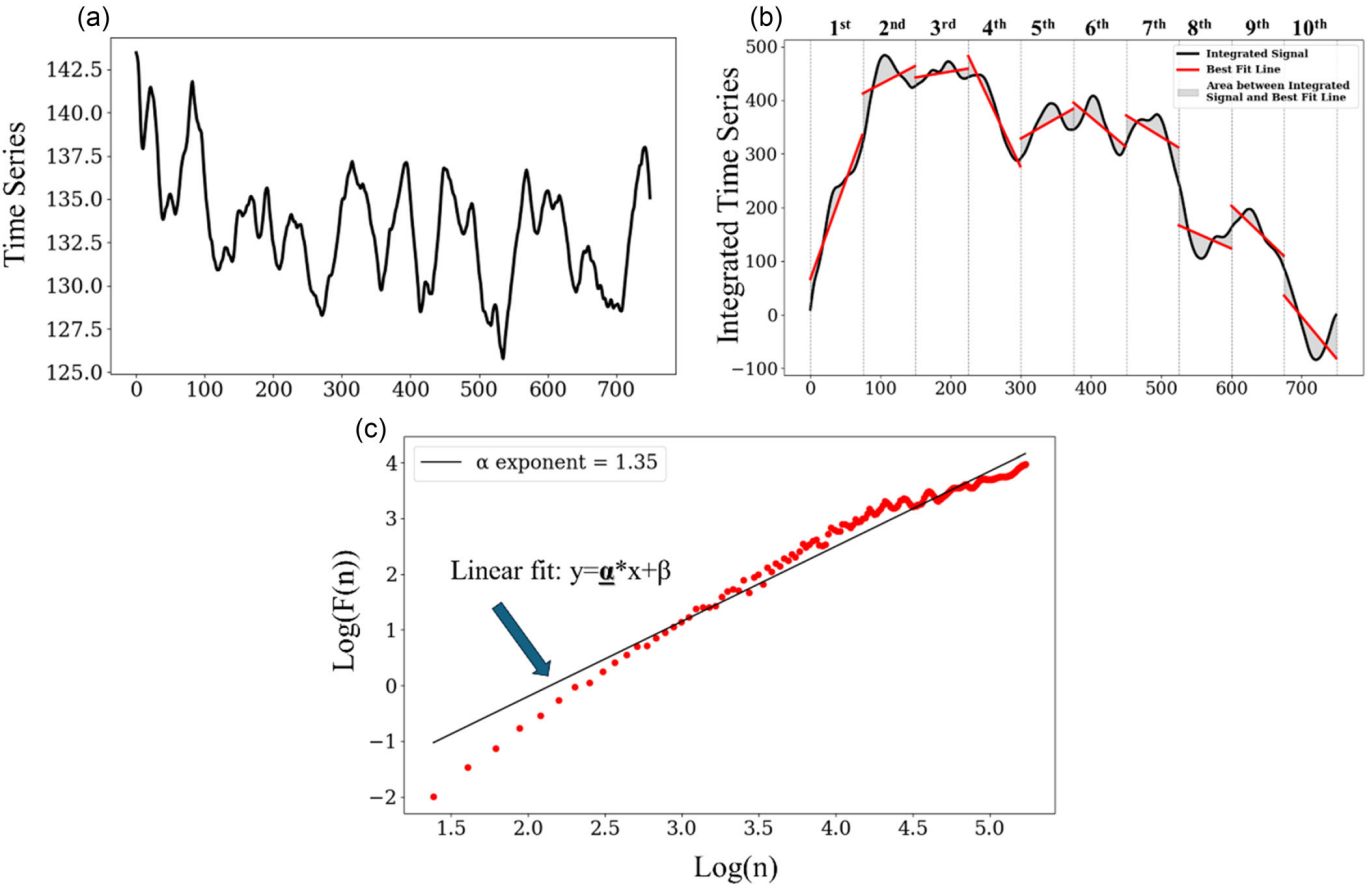
In the upper left graph, a time series is presented (a). In the upper right graph, the same time series is integrated (b). The integrated data is separated into 10 non‐overlapping windows, and the linear fit of each window is presented (b). In the lower graph, we calculate the slope of the linear fit of the Log(*F*(*n*)) to Log(*n*) (c).

The current secondary analysis was specifically designed to evaluate postural sway complexity using detrended fluctuation analysis (DFA) and multi‐scale entropy (MSE) as predefined outcomes. Complexity of the temporal structure of the postural sway variability was quantified with the alpha (α) exponent, derived from the DFA algorithm, which reflects the persistence of a time series [58]. The CoP time series (Figure 1a) is integrated and divided into windows of *n* size (Figure 1b). Then the data points of each window are fitted in a least square fit line; integrated data are then detrended by subtracting them from the fit line (Figure 1b). The root mean square is calculated for each window and summed across the whole time series, *F*(*n*). This process is repeated for other windows with progressively smaller *n* sizes. After the iteration of this process for all window sizes, the log(*F*(*n*)) is plotted against the log(*n*) and a linear fit is applied (Figure 1c). The slope of the linear fit is the α exponent (Figure 1d). The range of time scales (window sizes) that was selected for this analysis was 4 to *N*/4, where *N* corresponds to the total samples of the time series. The above calculation was performed separately for anterior‐posterior (AP) and mediolateral (ML) axes. A low α exponent indicates that the original time series exhibits greater randomness compared to a time series with a higher α exponent. As the CoP displays Brownian motion, the DFA values are typically high, ranging from 1.3 to 1.5 for young and healthy adults, while older individuals exhibit more random CoP movement with DFA values ranging from 1.1 to 1.3 [17, 23, 40, 44]. No previous DFA results, however, have been published with post‐TKA time as the independent variable.

**Figure 2:**
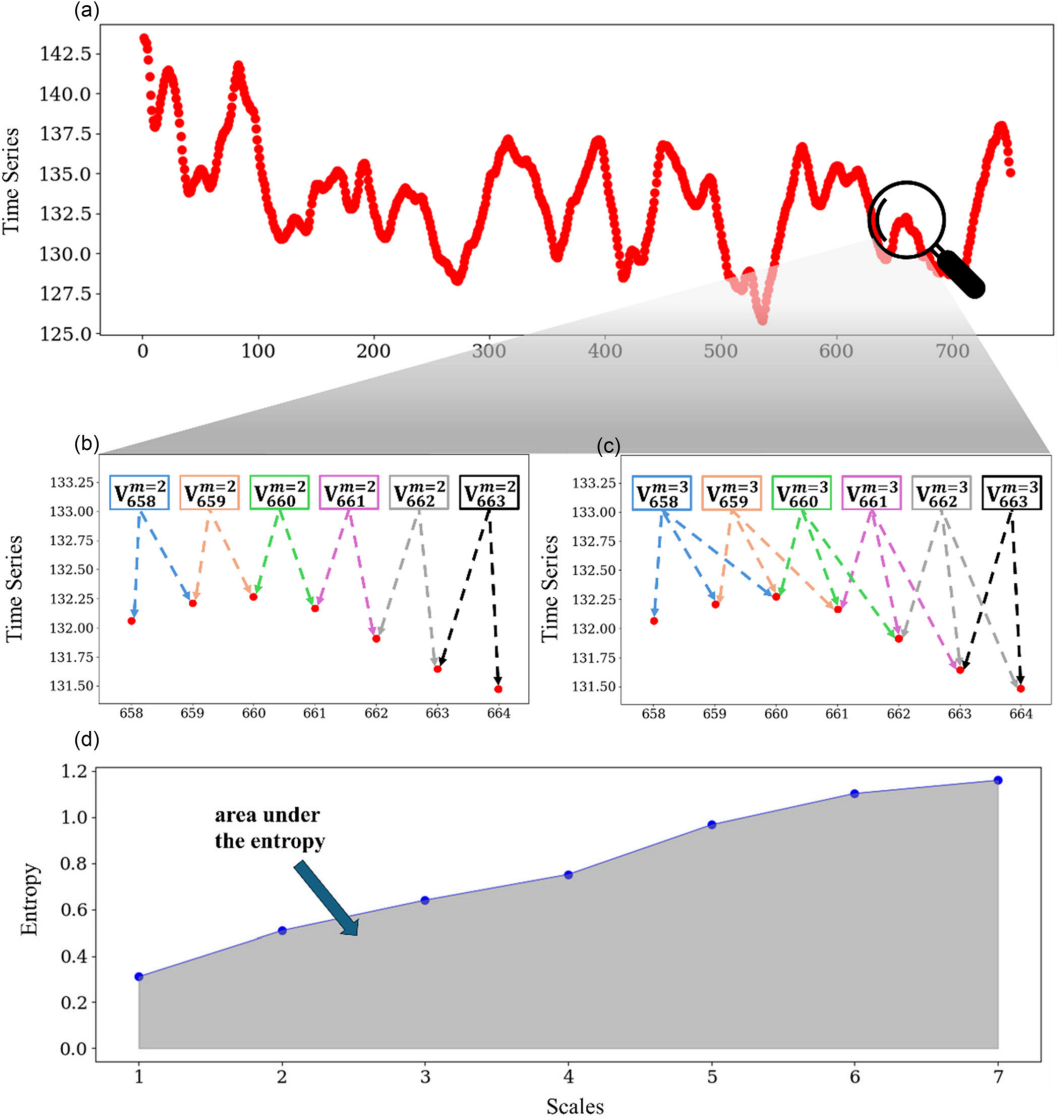
In the upper graph, a data series is presented (a). In the middle‐left graph, a portion of the upper graph is depicted, where the vectors for *m* = 2 are shown (b). In the middle right graph, the same portion is depicted, where the vectors for *m* = 3 are shown (c). In the lower graph, the sample entropy with tolerance *r* = 0.2 is presented for each time scale (d). Complexity of postural sway variability is defined as the area under the sample entropy to scale curve.

Additionally, the complexity of postural sway variability was quantified using MSE, which is a widely used technique that quantifies the probability or recurrence in physiologic series over different temporal or spatial scales [87]. In our case, the CoP series was ‘coarse‐grained’ for scales from 1 to 7. The original series was divided into non‐overlapping windows of length equal to 1–7 sampling points. In the coarse‐graining process, at Scale 1, we used the original series with 750 data points (Figure 2a), and at Scale 7, we averaged every 7 separate points, which gave us a new time series with 107 data points (750 points divided by 7). The sample entropy of each ‘coarse‐grained’ series was then calculated by using the negative natural logarithm of the conditional probability that a time series, having repeated itself within a tolerance *r* = 0.2 for *m* = 2 points (Figure 2b (pattern length)), will also repeat itself for *m* + 1 points without self‐matches (Figure 2c) [14, 35, 75]. Complexity of postural sway variability was then defined as the area under the entropy‐to‐scales curve, across seven scales (Figure 2d). Higher values of MSE (meaning a larger area under the curve) indicate that the original time series exhibits greater randomness compared to a time series with lower values of MSE. However, using MSE to quantify the complexity of postural sway variability, reference data of healthy movement are necessary. Therefore, differences in recording time and scales used produce non‐comparable results. Despite that, literature typically shows MSE values to be larger for older compared to younger adults [17, 23] and for fallers compared to non‐fallers [96].

### Statistical analysis

All statistical analyses were performed using JASP statistical software (version 0.19.3). Normality of distribution was verified using the Shapiro–Wilk test. The analysis was divided into two parts to address the distinct objectives of the study.

First, we used repeated measures analysis in the TKA patients to evaluate the effects of surgical approach (PP vs. MV) and postoperative time on postural sway complexity. We used a *2*_*b*_ *× 4*_*w*_ ANOVA with one between‐subjects factor (group: PP and MV) and one within‐subjects factor (Time: Pre, Post, 2 weeks and 1 month). Effect sizes were calculated as partial eta squared (*η*_*p*_^2^). When a significant main effect of time or interaction was observed, a set of planned contrasts was applied to further explore the differences. Specifically, Helmert contrasts were used to examine the effect of time by comparing each time point to the mean of subsequent time points. Effect sizes for the contrast comparisons were calculated using Cohen's *d*. For this analysis, the level of significance was set to 0.05.

Second, we conducted four separate one‐way ANOVAs to compare the postural sway complexity between the three groups at each point in time. This allowed the assessment of whether and when the TKA groups deviated from the normative values of the healthy control group. To account for multiple comparisons across the four time points, a Bonferroni‐corrected significance threshold of *p* < 0.0125 (0.05/4) was applied. Post hoc pairwise comparisons were performed using Tukey's honestly significant difference where appropriate, and effect sizes were reported using Cohen's *d*.

## RESULTS

### Repeated measures analysis in TKA patients

**Figure 3:**
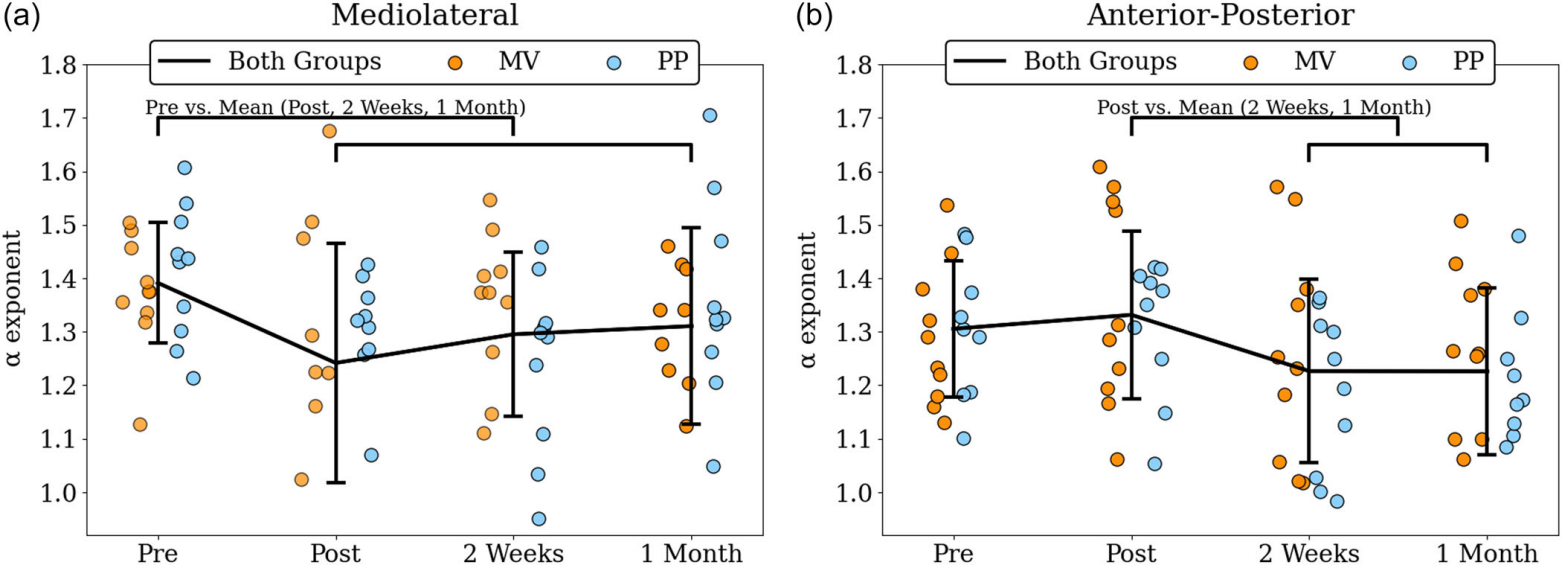
α exponent of CoP at the mediolateral (a) and the anterior‐posterior (b) axis for both groups (black line), and separately for MV (orange dots) and for PP (blue dots) for all measured timepoints. Annotated brackets indicate the statistically significant contrasts. CoP, center of pressure; MV, mid‐vastus; PP, parapatella.

Analysis was performed on the CoP sway time series for each axis of movement, the ML and the AP. Regarding the α exponent in the ML axis, no significant interaction (*F*_(3,54)_ = 1.448, *p* = 0.239, *η*_*p*_^2^ = 0.074) or group effect was observed (*F*_(1,18)_ = 0.125, *p* = 0.728, *η*_*p*_^2^ = 0.007). However, a significant effect of time was observed (*F*_(3,54)_ = 3.022, *p* = 0.037, *η*_*p*_^2^ = 0.144). Further investigation of the effect of time using contrasts revealed that the α exponent in the Pre was significantly larger than all the subsequent time points (*t*_(18)_ = 3.166, *p* = 0.005, *d* = 0.611). α exponent in the Post was not significantly different from the subsequent time points (*t*_(18)_ = −1.216, *p* = 0.240, *d* = −0.344) and α exponent in the 2 weeks was not different compared to the 1 month (*t*_(18)_ = −0.310, *p* = 0.760, *d* = −0.086; Figure 3a).

No significant interaction (*F*_(3,54)_ = 1.067, *p* = 0.371, *η*_*p*_^2^ = 0.056) or group effect was also observed (*F*_(1,18)_ = 0.606, *p* = 0.447, *η*_*p*_^2^ = 0.033) for α exponent in the AP axis as well. A significant effect of time was again observed (*F*_(3,54)_ = 4.340, *p* = 0.008, *η*_*p*_^2^ = 0.194). Further investigation of the effect of time using contrasts revealed that the α exponent in the Pre was not significantly different from the subsequent time points (*t*_(18)_ = 1.619, *p* = 0.123, *d* = 0.277). α exponent in the Post was significantly larger than the subsequent time points (*t*_(18)_ = 3.148, *p* = 0.006, *d* = 0.664), and the α exponent in the 2 weeks was not different compared to the 1 month (*t*_(18)_ = −0.310, *p* = 0.760, *d* = 0.086; Figure 3b).

**Figure 4:**
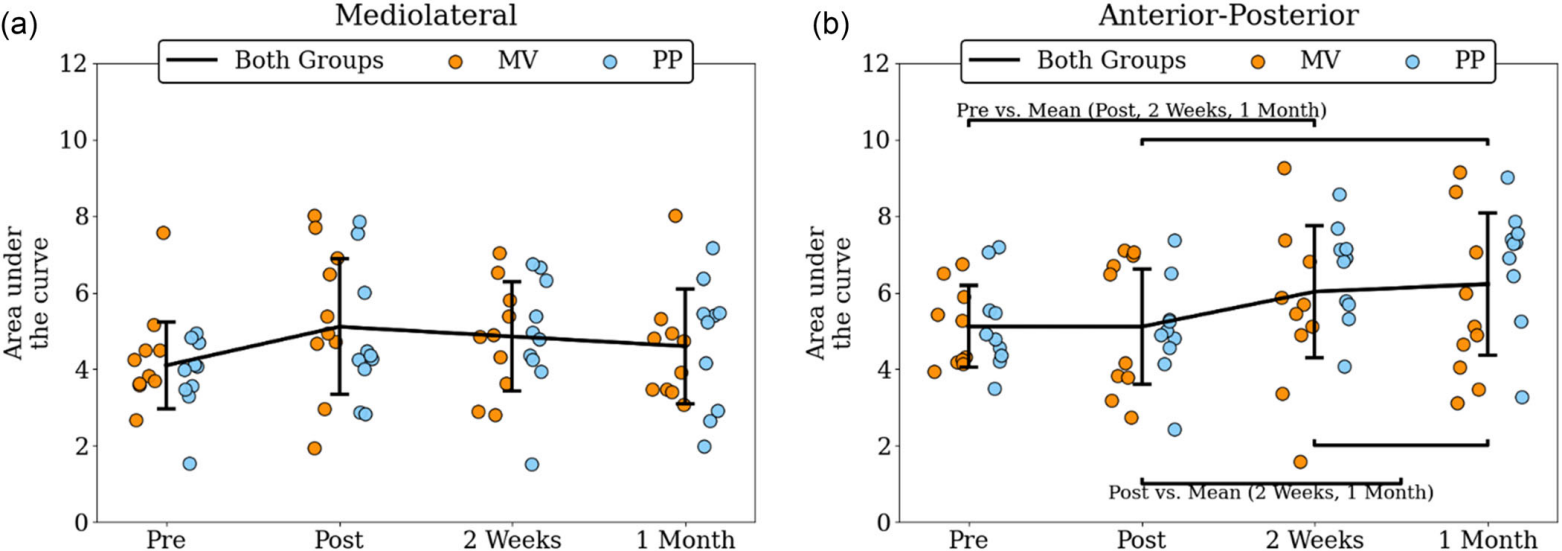
MSE of CoP at the mediolateral (a) and the anterior‐posterior (b) axis for both groups (black line), and separately for MV (orange dots) and for PP (blue dots) for all measured timepoints. Annotated brackets indicate statistically significant contrasts. CoP, center of pressure; MSE, multi‐scale entropy; MV, mid‐vastus; PP, parapatellar.

Regarding the MSE in the ML axis, no significant interaction (*F*_(3,54)_ = 0.405, *p* = 0.750, *η*_*p*_^2^ = 0.022), group (*F*_(1,18)_ = 0.157, *p* = 0.697, *η*_*p*_^2^ = 0.009), or time effects was observed (*F*_(3,54)_ = 2.291, *p* = 0.089, *η*_*p*_^2^ = 0.113) (Figure 4a). Regarding the AP axis, no significant interaction (*F*_(3,54)_ = 1.361, *p* = 0.265, *η*_*p*_^2^ = 0.070) or group effect was observed (*F*_(1,18)_ = 1.037, *p* = 0.322, *η*_*p*_^2^ = 0.054). However, a significant main effect of time was observed (*F*_(3,54)_ = 4.225, *p* = 0.009, *η*_*p*_^2^ = 0.190). Further investigation of the effect of time using contrasts revealed that MSE in the Pre was significantly smaller than the subsequent time points (*t*_(18)_ = −2.573, *p* = 0.019, *d* = −0.422). MSE in Post was significantly smaller than the subsequent time points (*t*_(18)_ = −2.573, *p* = 0.025, *d* = −0.636; Figure 4b).

### Comparison with controls

**Figure 5:**
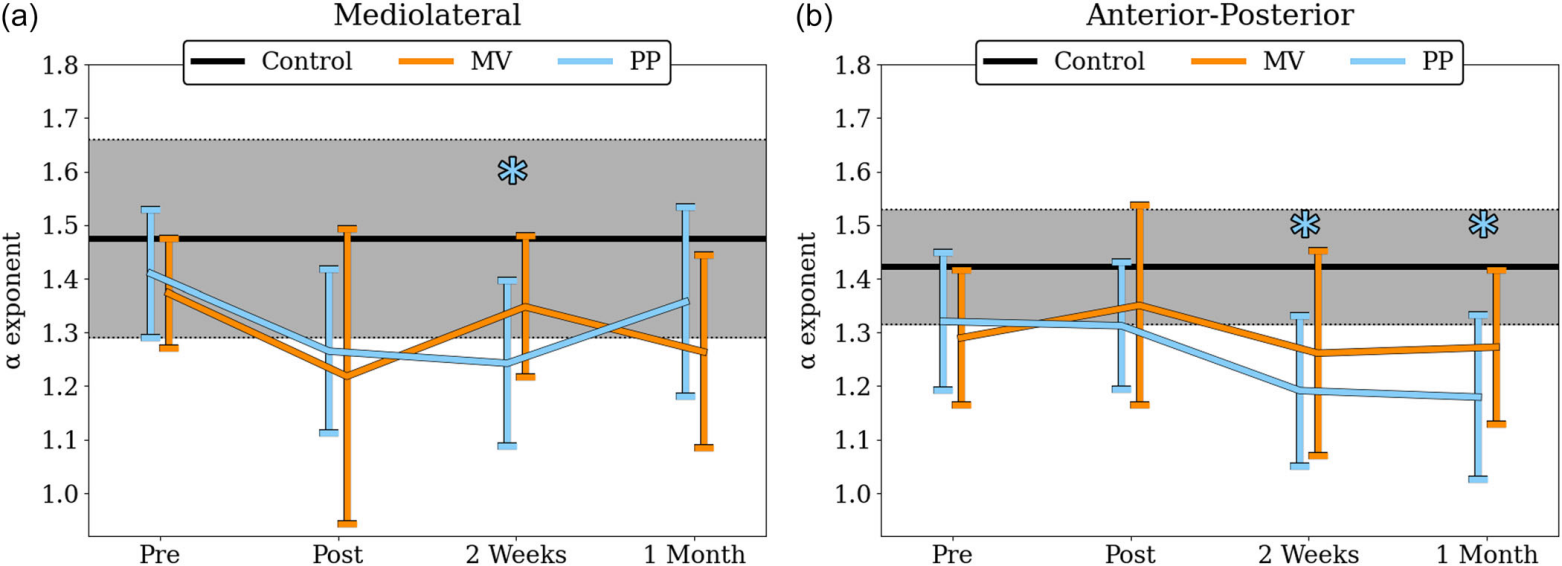
α exponents of control (black), PP (blue) and MV (orange) groups for the mediolateral (a) and the anterior‐posterior (b) axes. Asterisks (*) indicate statistically significant differences between the PP and control groups. MV, mid‐vastus; PP, parapatellar.

**Figure 6:**
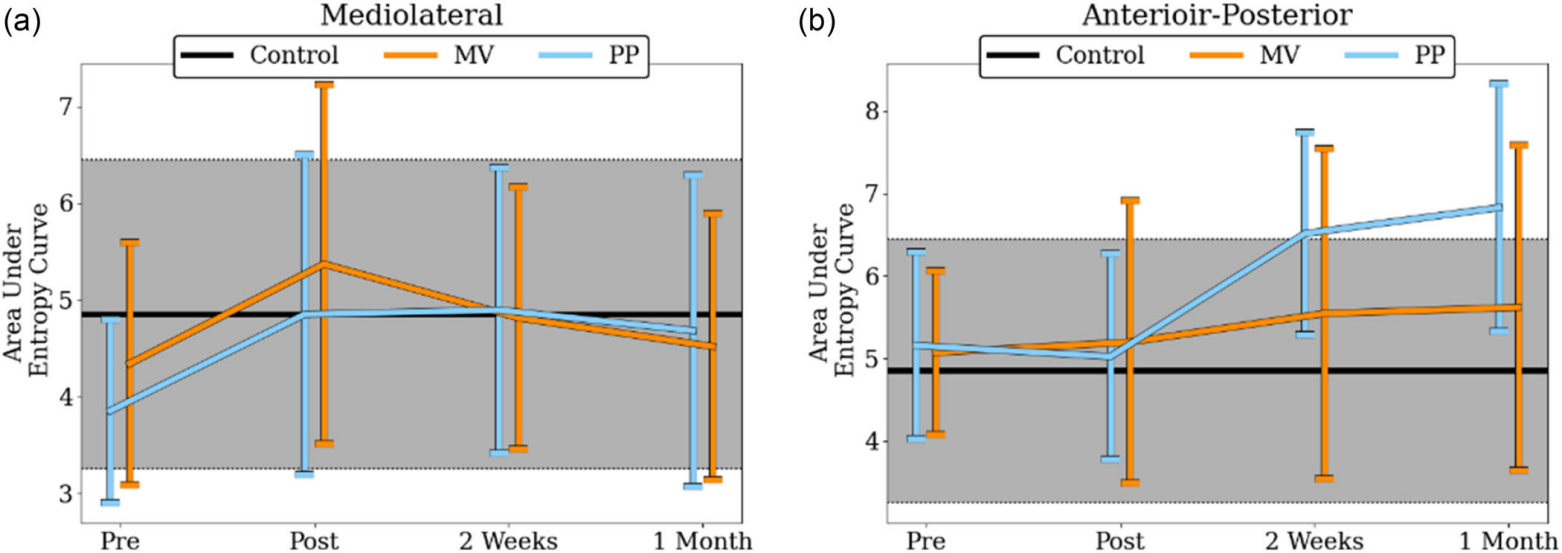
MSE of control (black), PP (blue) and MV (orange) groups for the mediolateral (a) and the anterior‐posterior (b) axes. MSE, multi‐scale entropy; MV, mid‐vastus; PP, parapatellar.

Regarding the α exponent of postural sway for the ML axis, a significant difference was noticed between the three groups only at 2 weeks (*F*_(2,28)_ = 5.090, *p* = 0.012, *η*_*p*_^2^ = 0.266). Post hoc test indicated that α exponent was significantly lower for the PP group compared to the Control group (*p* = 0.010, *d* = 1.388; Figure 5a). Regarding the α exponent of postural sway for the AP axis, a significant difference was noticed between the three groups at 2 weeks (*F*_(2,28)_ = 6.002, *p* = 0.007, *η*_*p*_^2^ = 0.300) and 1 month (*F*_(2,28)_ = 7.783, *p* = 0.002, *η*_*p*_^2^ = 0.357). Post hoc test indicated that α exponent was significantly lower for the PP group compared to the Control group at both 2 weeks (*p* = 0.006, *d* = 1.468) and 1 month (*p* = 0.002, *d* = 1.703; Figure 5b). No significant differences in MSE were observed between the three groups at any of the four time points, nor across both axes (Figure 6a,b).

## DISCUSSION

The present study aimed to identify how the complexity of postural sway variability is altered in patients who undergo TKA and whether these changes differ by surgical approach. More specifically, we examined the acute effects of a traditional (PP) and a minimally invasive (MV) TKA approach on the complexity of postural sway variability. We hypothesized that both patient groups would demonstrate reduced sway complexity following TKA compared to pre‐surgery levels and that patients who undergo TKA using the PP approach would demonstrate greater reduction in sway complexity compared to patients who undergo TKA using the MV approach. In addition, we compared both TKA groups to a healthy control group at each time point to contextualize postural sway complexity relative to normative postural control. We hypothesized that there would be no significant differences between the PP and MV groups prior to surgery, and that both groups would exhibit more random patterns compared to the control group. Furthermore, we expected that during recovery, the PP group would demonstrate greater deviations from the healthy control group than the MV group. The first hypothesis was confirmed, as the α exponent in both axes of motion and the MSE in the AP axis were significantly lower and higher following the surgery, respectively, compared to Pre surgery values. These findings suggest a disruption in the 1/*f* structure of the CoP sway, indicating a shift towards more random dynamics. Although differences between the PP and MV groups were not statistically significant in the repeated measures analysis, the PP group exhibited a trend towards lower α exponent and increases in MSE, suggesting more pronounced loss of complexity. Finally, when compared to healthy controls, significant deviations in the α exponent were observed only in the PP group, further supporting the notion that this surgical approach may lead to greater disruption in the temporal structure of postural sway variability.

Loss of complexity is often reported in the literature for aged and pathologic populations [30, 45, 76], which may provide an explanation for the reduction in complexity which was observed following TKA in this study. The α exponent values observed in this study are comparable with those previously reported for older adults [17, 22]. In addition, the decreased values of the α exponent and the increased values of the MSE both metrics indicate increased randomness in the CoP sway variability and a loss of complexity [17, 23]. While our previous study showed increased postural sway magnitude for the PP group [55], the current analysis extends these results by identifying a greater loss of complexity for the same group, a stronger predictor of future fallers [96]. The present analytical approach, therefore, offers a more robust indicator for assessing acute effects of TKA where the risk of falling is high [5, 10, 11].

Comparison of postural sway complexity between TKA patients and healthy controls revealed that the α exponent of the PP group exhibited significant deviations in the ML axis at 2 weeks (Figure 5a) and in the AP axis at 2 weeks and 1 month (Figure 5b). The different ways the two surgical approaches act upon the treated knee should account for the different amount of complexity loss following TKA. Based on the findings of Zhou et al. [96], who linked reduced complexity with increased fall risk, our findings suggest that patients in the PP group may be at greater risk of future falls, consistent with Pethes et al., who reported similar outcomes in TKA patients undergoing postural perturbation testing. The PP group showcased decreased adaptive capacity compared to the minimally invasive group, and the authors suggested that PP surgically treated patients may be more prone to falling 12 weeks after the surgery [59]. Similar conclusions were drawn when gait stability was assessed in both the traditional and minimally invasive TKA groups [41]. Although MSE did not reveal statistically significant group differences, its mean values tended to increase postoperatively, particularly in the AP axis. This directional shift is consistent with the DFA findings, both suggesting a move towards a more random temporal structure following TKA. The discrepancy in statistical significance may be due to the two algorithms capturing different aspects of the signal. The α exponent reflects the persistence of the signal meaning its tendency to retain its behavioural direction, whereas MSE quantifies the irregularity of the signal by quantifying the unpredictability of future values. Future studies should investigate whether such discrepancies are purely mathematical or can reveal meaningful insights into postural sway variability.

A possible explanation for the reduced complexity in the PP group could be that the PP approach imposes greater damage in the overall joint area, leading to reduced knee functionality that includes reduced flexion and extension strength [3, 55, 78, 81], reduced range of motion [2, 46] and increased postural sway [13, 55, 73] compared to the MV approach. The more substantial mechanical constraints imposed by the PP approach may therefore contribute to the more pronounced loss of postural sway complexity observed in this group. Interestingly, however, the postural sway complexity of the PP group was not different from healthy controls 5 days after the surgery (Figure 5b). Considering that patients remain hospitalized and do not stand or walk unassisted for up to five days after TKA, it is likely that they have not yet started adapting to the new anatomical constraints introduced by the surgery. The temporal structure of variability, as reflected in postural sway complexity, represents the self‐organization process of the human system to solve a given motor task while accounting for both anatomical‐biomechanical and task‐environmental constraints [29, 76]. In our study, PP patients must solve the quiet standing task given different anatomical constraints than MV patients. We conclude that this stronger constraint is leading to a less complex, stochastic variability pattern that is formed after the first five days, when the patients begin to perform the quiet standing task. Finally, the same trend is also present 1 month after the surgery. According to Clark et al., α values were found to be higher at 12 weeks compared to 4 weeks post‐TKA, suggesting that postural sway complexity may be restored within approximately three months [13]. If, however, early‐stage stochastic patterns in postural sway are correlated with falls in TKA patients, as observed in older adults [86, 96], conclusions can be drawn about the prediction of fallers and the potential development of targeted interventions for patients at risk.

The loss of postural sway complexity after TKA has significant implications. Our data, to our knowledge, are the first to suggest a connection between postural sway complexity and TKA‐induced deterioration in postural control. Given that loss of complexity in postural sway is associated with an increased risk of falling [86, 96], we propose a new analytical approach that could potentially be used to prevent falls during the post‐TKA period. Although our findings cannot be directly linked to future fall predictions, they offer preliminary knowledge for future experiments. The next logical steps in research should involve acute evaluation of postural complexity as well as longitudinal observation of patients. This line of research could eventually lead to the development of clinical tools that rehabilitation professionals can use to tailor interventions based on each patient's risk of falling. Still, we acknowledge that statistically significant results do not always imply clinical benefit, and future studies must determine whether changes in sway complexity represent meaningful improvements in patient outcomes [56]. Furthermore, the analytical approach presented here could also be applied to athletic populations, where alterations in postural sway complexity (e.g., following concussion or ACL injury) have been shown to persist even after traditional performance metrics return to normal [53, 63, 64].

This study has some limitations. First, a larger sample size is needed to draw definitive conclusions about loss of complexity in TKA. Another limitation of our protocol is that patients were monitored only during the first month of rehabilitation. In future studies, we plan to follow patients longitudinally in a fully blinded randomized control trial and investigate whether postural sway complexity returns to normal values at some point in time after TKA. Longitudinal observations should also be performed in healthy older adults. This way, the reduction in postural sway complexity due to ageing will be considered. Such longitudinal observation should also be accompanied by functional tests and performance and functionality questionnaires to better describe the post‐operative status of the patients and correlate it with postural sway complexity. Monitoring fall incidents may also help to better understand the connection between postural sway complexity and the adaptive capacity of patients. Finally, although our control group provided age‐ and sex‐matched normative data, the use of publicly available data may introduce within‐group variance due to procedural or demographic differences.

## CONCLUSION

In this study, we provide preliminary evidence for the loss of postural sway in women undergoing TKA, with the PP approach resulting in greater loss compared to the MV approach. Patients in the PP group showed significantly lower α exponent values at 2 weeks and 1 month, suggesting a shift towards a more random temporal structure of postural sway variability. No such significant deviations were observed for the MV group. This study is a first step towards identifying postural sway complexity in the short‐term post‐TKA period. Given the link between random postural sway structure and increased fall risk, our results suggest that such an approach may serve as a candidate biomarker for identifying patients at higher risk of falling after TKA. We plan on extending this line of research by addressing the current study's limitations to implement the metrics of our study as indicators of adaptive capacity after TKA, with the eventual goal of predicting better surgical outcomes.

## AUTHOR CONTRIBUTIONS

**Vasileios Mylonas**: Conceptualization; writing—original draft; software; methodology; formal analysis. **Stylianos Grigoriadis**: Conceptualization; writing—review and editing; software; methodology; formal analysis. **Dimitris Metaxiotis**: Writing—review and editing; data curation; project administration. **Eleftherios Kellis**: Writing—review and editing; project administration. **Nick Stergiou**: Conceptualization; writing—review and editing; formal analysis; supervision. **Thomas Nikodelis**: Conceptualization; project administration; writing—review and editing; supervision.

## CONFLICT OF INTEREST STATEMENT

The authors declare no conflicts of interest.

## ETHICS STATEMENT

The ethics statement is not available.

## Supporting information

STROBE Checklist.

## Data Availability

The data sets analyzed during the current study are available from the corresponding author on reasonable request.
