# Supplementary material for: Medial parapatellar surgical approach leads to greater loss of postural sway complexity compared to mid‐vastus approach in women undergoing total knee arthroplasty
Source: Knee Surg Sports Traumatol Arthrosc. 2025 Sep 9;34(5):1700–12. doi: 10.1002/ksa.70057 (PMC13122746; doi:10.1002/ksa.70057)
Supplement: Supplementary file 1 — STROBE Checklist. [file KSA-34-1700-s001.docx]

| **STROBE Item** | **Description** | **Addressed in Manuscript** |
| --- | --- | --- |
| **1. Title and Abstract** | Study design ('exploratory comparative analysis') is stated in the abstract and clarified in the added STROBE paragraph. | Title - Abstract |
| **2. Background/Rationale** | Describes the need to assess postural sway complexity after TKA and prior gaps in literature. | Introduction |
| **3. Objectives** | States three hypotheses regarding TKA approaches and postural sway complexity. | Introduction |
| **4. Study Design** | Exploratory comparative observational design using secondary analysis with a control group. | Abstract - Methods |
| **5. Setting** | Data collected at a hospital in a previous study along with a publicly available control dataset; described in Participants section. | Methods - Participants |
| **6. Participants** | Eligibility, group allocation, demographics, and randomization method described. | Methods - Participants |
| **7. Variables** | Defines Hurst exponent and MSE as complexity measures (primary outcomes). | Methods - Data Analysis |
| **8. Data Sources / Measurement** | Force plate recordings and processing detailed; downsampling justified; source of control data cited. | Methods - Data Analysis |
| **9. Bias** | Blinding and allocation concealment were not performed and acknowledged; limitation acknowledged. | Methods - Discussion |
| **10. Study Size** | Sample size was limited; no power analysis due to exploratory nature; limitation acknowledged. | Limitations |
| **11. Quantitative Variables** | Details given for computation of DFA/MSE and comparison strategy using ANOVA. | Methods - Data Analysis |
| **12. Statistical Methods** | Repeated measures ANOVA, post hoc tests, effect sizes, and Bonferroni correction described. | Methods - Statistical Analysis |
| **13. Participants** | Describes total included participants, grouping, and demographics. | Methods - Participants |
| **14. Descriptive Data** | Participant demographics and group characteristics reported. | Methods - Participants |
| **15. Outcome Data** | DFA and MSE results reported across time points and groups; visualized in figures. | Results |
| **16. Main Results** | Group and time effects reported with statistics, p-values, and interpretation. | Results |
| **17. Other Analyses** | Post hoc comparisons between surgical groups and controls reported. | Results |
| **18. Key Results Summary** | Summarizes key findings linking surgical approach to loss of complexity. | Discussion |
| **19. Limitations** | Sample size, follow-up duration, lack of blinding noted. | Methods - Discussion |
| **20. Interpretation** | Balanced discussion of implications, potential biomarker value, and caution around claims. | Discussion - Conclusion |
| **21. Generalizability** | Cautions about sample size and sex-specific focus; suggests future validation. | Discussion - Conclusion |
| **22. Funding** | States funding sources and no conflicts of interest. | Declarations |
